# Assessment of [^125^I]a-Bungarotoxin Binding to a7 Nicotinic Acetylcholinergic Receptors in Hippocampus-Subiculum of Postmortem Human Parkinson’s Disease Brain

**DOI:** 10.3390/biom15121686

**Published:** 2025-12-02

**Authors:** Fariha Karim, Allyson Ngo, Titus E. Tucker, Ashlee D. L. Coronel, Jogeshwar Mukherjee

**Affiliations:** Preclinical Imaging, Department of Radiological Sciences, University of California-Irvine, Irvine, CA 92697, USA; fkarim1@uci.edu (F.K.); allyson1@uci.edu (A.N.); tetucker@uci.edu (T.E.T.); adcorone@uci.edu (A.D.L.C.)

**Keywords:** [^125^I]a-bungarotoxin, nicotinic acetylcholinergic receptors, Parkinson’s disease, ubiquitin, α-synuclein, Lewy body, [^18^F]ASEM

## Abstract

Parkinson’s disease (PD) involves motor and cognitive impairment that nicotinic acetylcholine receptors (nAChRs) such as the α7 subtype are responsible for regulating. The hippocampus, abundant in α7 nAChRs, was quantitatively evaluated for [^125^I]α-bungarotoxin ([^125^I]α-Bgtx) binding to α7 nAChRs in postmortem human PD (n = 26; 12 male, 14 female) and cognitively normal (CN) (n = 29; 14 male, 15 female) brain slices. Anti-ubiquitin and anti-α-synuclein immunostained adjacent slices were analyzed using QuPath. Autoradiographs of [^125^I]α-Bgtx radioligand binding were analyzed in OptiQuant. Ubiquitin and α-synuclein distribution generally aligned with the distribution of α7 nAChRs detected by [^125^I]α-Bgtx. Binding of [^125^I]α-Bgtx in PD cases was significantly greater than CN with a 32% increase in gray matter binding. A weak positive correlation between age and [^125^I]α-Bgtx binding was found in both PD and CN. In comparison to Alzheimer’s disease hippocampus, [^125^I]α-Bgtx binding in PD gray matter was higher by 41%. Differences in nAChR expression imply unique roles depending on the neurodegenerative pathology. PD may experience an increase in α7 nAChRs as a compensatory mechanism to the loss in neurons, highlighting its neuroprotective capabilities. [^125^I]α-Bgtx shows potential as a radioligand for α7 nAChRs to elucidate the complexities of PD pathology.

## 1. Introduction

**Figure 1:**
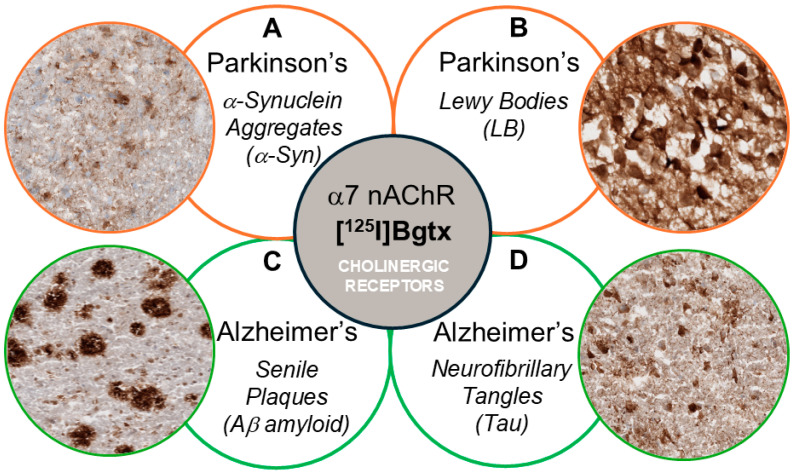
Schematic showing potential effects on α7 nAChRs labeled with [^125^I]α-Bgtx. (**A**) a-Synuclein aggregates may affect [^125^I]α-Bgtx binding to α7 nAChRs in PD. (**B**) Lewy bodies’ accumulation and cell death affect [^125^I]α-Bgtx binding to α7 nAChRs in PD. (**C**) Ab plaques did not affect [^125^I]α-Bgtx binding to α7 nAChRs in postmortem AD hippocampus [9]. (**D**) [^125^I]α-Bgtx binding was unaffected by tau (neurofibrillary tangles, NFTs) in postmortem AD hippocampus [9].

Parkinson’s disease (PD) is a neurodegenerative disorder that involves impairment in motor and cognitive capabilities [1]. The pathology of PD is characterized by the progressive degeneration of dopamine neurons that may involve Lewy bodies composed of α-synuclein aggregates [2]. Lewy body formation is a major contributor to neurodegeneration in PD in addition to α-synuclein fibrillization [3]. The abnormal misfolding of α-synuclein can damage neurons and cause mitochondrial dysfunction, contributing to neurodegeneration in PD [4,5]. This mitochondrial dysfunction includes the mitochondria surrounding Lewy bodies; therefore each dysfunctional protein including mitochondria is identified to be marked for degradation by ubiquitin. However, aggregation of ubiquitinated proteins within Lewy bodies overwhelms the ubiquitin-proteasome degradation system and ubiquitinated proteins accumulate instead of undergoing degradation [6]. Ubiquitin is one of the most accurate markers for Lewy bodies throughout numerous tissue and cell types [7,8]. Both α-synuclein aggregates and Lewy bodies are prominent in PD pathophysiology (Figure 1).

As a neurodegenerative disease primarily identified by motor symptoms, non-motor symptoms may also contribute to PD pathology. Dopaminergic neuron degeneration in the nigrostriatal dopaminergic pathway is the primary neuropathological attribution to PD motor impairment [1]. Non-motor symptoms arise from the spread of α-synuclein and disrupted neurotransmitter systems such as serotonin and acetylcholine [3]. In the striatum where there are high levels of dopamine and acetylcholine, neurotransmitter systems can interact and establish responses related to motor and cognitive functions [10]. Cholinergic impairments independent of dopaminergic degeneration have been linked to PD symptoms including gait dysfunction, levodopa-induced dyskinesias, and dementia [10,11]. Nicotinic acetylcholine receptors (nAChRs) have diverse functions as ligand-gated ion channels including the regulation of physiological processes such as memory, cognition, and motor abilities. Among the numerous nAChR subtypes, α7 nAChRs are homomeric pentamers that are crucial for maintaining neurotransmission and neuroprotection in neurons throughout the central nervous system. Expression of α7 nAChR is abundant in brain regions that contribute to the cholinergic system for cognitive function such as cerebral cortex and hippocampus [12]. Potentiated by strong calcium influxes, many regulatory functions of α7 nAChRs such as neuroprotective and anti-inflammatory effects are impacted by multiple neurological diseases including PD [13]. PD dementia has a complicated physiopathology with severe dopaminergic and cholinergic deficits that involves synergy between α-synuclein aggregates and biomarkers of Alzheimer’s disease (AD) pathology [10]. Thus, there has been a strong interest in identifying the effect of the various pathologies in PD and AD on α7 nAChR (Figure 1).

**Figure 2:**
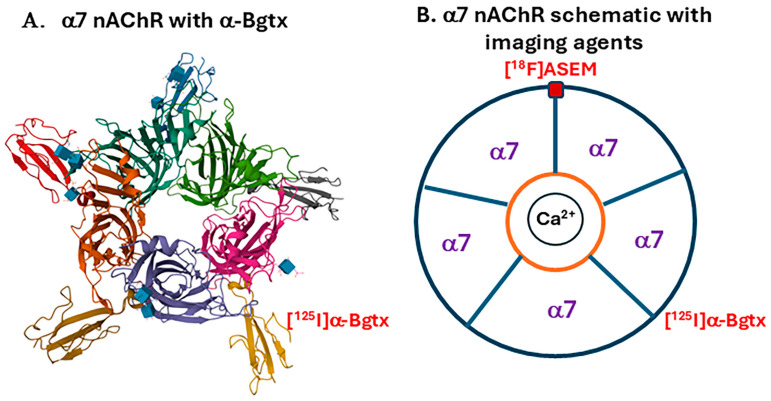
α7 nAChRs imaging agents (**A**). α7 nAChR subunit structures complexed with α-Bgtx [19]. Five molecules of α-Bgtx surround the α7 nAChR, binding between α7 subunits. The α-Bgtx molecules are colored in yellow, brown, red, blue, and gray while the inner molecules represent α7 subunits (**B**). Schematic showing binding [^125^I]α-Bgtx (for in vitro studies) and [^18^F]ASEM (for PET studies) to α7 nAChR subunits. The red rectangle is an acetylcholine site where [^18^F]ASEM binds. [^125^I]α-Bgtx binds at the interface between α7 subunits. In the middle of the schematic is calcium (Ca^2+^) to represent the passage of Ca^2+^ ions that result from the activation of α7 nAChRs.

[^125^I]a-iodobungarotoxin ([^125^I]a-Bgtx) was shown to selectively bind to α7 nAChRs and has been widely used [14,15,16]. Similarly to previous reports for a7 nAChRs, our results with a-[^125^I] Bgtx in rats and mice brains gave ratios versus cerebellum as a reference region in the following order: hippocampus> inferior colliculus> frontal cortex> superior colliculus> subiculum> thalamus> striatum> cerebellum [17]. When comparing the ratios of α7 nAChRs in mice and rat brains, there were minor differences in the inferior and superior colliculi where they both observed a discreet presence of α7 nAChRs. Using α7 nAChR gene-deficient mice, selectivity of [^125^I]a-Bgtx for α7 nAChRs was demonstrated in vitro [18]. The structure of the homomeric pentameric α7 nAChR complex with α-Bgtx was reported [19]. The C-terminus of [^125^I]a-Bgtx is lodged into loop C of the α7 nAChR binding site (Figure 2A). [^125^I]a-Bgtx binds at the interface between α7 subunits on the nAChR.

Visualization of α7 nAChRs has been investigated by developing radioligands to use positron emission tomography (PET) imaging. One of the first successful PET radioligands to demonstrate high specificity to human α7 nAChRs is [^18^F]ASEM [20,21,22]. ASEM has high binding affinity for α7 nAChRs as an antagonist, visualized in humans by synthesizing ASEM as a ^18^F-labeled probe. [^18^F]ASEM has been used to investigate α7 nAChRs in a variety of conditions such as skeletal muscle denervation in mice [23], atherosclerosis in humans [24], mild cognitive impairment (MCI) in humans [25], and psychosis [26,27]. Higher α7 nAChR availability was observed in MCI over controls while a lower α7 nAChR availability was observed in psychosis in [^18^F]ASEM PET studies. PET studies in a rat model of PD have shown an increase in [^18^F]ASEM in unilaterally 6-hydroxydopmaine lesioned rats [28]. Because a-[^125^I] Bgtx binding has been shown to be very similar to [^125^I]IASEM (an analog of [^18^F]ASEM) for α7 nAChRs [18], there is significant translational potential of a-[^125^I] Bgtx postmortem studies. In concordance with this concept, [^125^I]α-Bgtx has been successfully used in postmortem Alzheimer’s disease (AD) to study α7 nAChRs [9]. However, there are minimal observations of [^125^I]α-Bgtx in PD despite how the pathology may affected by cholinergic deficits involving α7 nAChRs. A nonhuman primate study with monkeys treated with 1-methyl-4-phenyl-1,2,3,6-tetrahydropyridine (MPTP) found increased [^125^I]α-Bgtx binding in the dorsolateral putamen [29]. In contrast, there are more reports on the loss of a4b2 nAChRs in PD [30,31,32]. Thus, although there are studies about the binding of numerous other nAChRs subtypes, autoradiographic studies using [^125^I]α-Bgtx to bind to α7 nAChRs in PD is currently lacking.

Human hippocampal α7 nAChRs constitute a vital neurotransmitter–receptor system that plays a role in cognition [33]. Pathologies and neuroinflammation found in PD may have an adverse effect on α7 nAChRs causing serious damage to cellular functions [34,35,36]. Thus, the aim of this study was to measure in vitro [^125^I]α-Bgtx binding to α7 nAChRs in postmortem hippocampal brain slices from PD cases and cognitively normal (CN) cases. Our previous findings in postmortem hippocampal brain slices from AD cases did not reveal significant changes compared to CN cases [9].

## 2. Materials and Methods

### 2.1. Postmortem Human Brain

Human postmortem brain tissue samples were obtained from Banner Sun Health Research Institute (BHRI), Sun City, AZ, USA, a brain tissue repository for in vitro experiments. Detailed characteristics of the obtained frozen brain samples were received from BHRI, Sun City, Arizona (Table 1). Brain tissue samples from CN and PD cases were selected by observing the presence and absence of end-stage pathology. All PD and CN cases were categorized with minimal pathologies of other diseases. AD cases were previously reported [9]. Brain slices were cut 10 mm thick using a Leica 1850 cryotome (Leica Biosystems, Deer Park, IL, USA) set to −20 °C. Cut brain slices were adhered to glass slides (Thermo Fisher Scientific Inc., Waltham, MA, USA). The brain slices contained the hippocampus region (CN, *n* = 29; ages 53–95 and PD, *n* = 26, ages 65–87; Table 1). Brain sections were stored at −80 °C. All postmortem human brain studies were approved by the Institutional Biosafety Committee of University of California, Irvine.

### 2.2. Radiopharmaceuticals

[^125^I]α-Bgtx (a-Bungarotoxin, Tyr54-^125^I, molecular weight ~8119) was purchased from American Radiolabeled Chemicals, Inc. (Saint Louis, MO, USA). The HPLC purified product was >98% radiochemical pure, with >95% chemical purity, and a molar activity of >70 GBq/µmol.

### 2.3. [^125^I]α-Bgtx Autoradiography

All postmortem human brain slices underwent autoradiography procedures as previously reported [9,17]. Postmortem human brain slices (10 mm) of the hippocampus adhered to Fisher glass slides were taken out of an −80 °C freezer and placed into glass chambers. All slides of brain slices were preincubated in 50 mM Tris HCl, pH 7.3, containing 0.1% bovine serum albumin (BSA) at room temperature for 30 min. After the preincubation, [^125^I]α-Bgtx was added to the glass chambers to incubate all the brain slices at room temperature for 120 min. After incubation, the slides were washed within their chambers with ice-cold Tris buffer, pH 7.3, three times for 10 min each. A quick wash of cold (0–5 °C) deionized water shortly followed. After air drying, the slides with brain sections were placed into a film cassette with a phosphor screen film. After a week, the film was taken out and inserted into the Phosphor Autoradiographic Imaging System/Cyclone Storage Phosphor System (Packard Instruments Co). The resultant autoradiographic image was opened on the OptiQuant acquisition and analysis program (Packard Instruments Co) to draw regions of interest and measure [^125^I]α-Bgtx binding in DLU/mm^2^.

### 2.4. Immunohistochemistry

Immunostaining with anti-ubiquitin and anti-a-synuclein of all brain slices was carried out by University of California-Irvine, Pathology services as previously described [32]. Slides that underwent immunohistochemistry (IHC) staining were scanned using the Ventana Roche instrumentation (Ventana Medical Systems, Oro Valley, AZ, USA) and analyzed using QuPath (version QuPath-0.4.2).

### 2.5. Statistical Analysis

The DLU/mm^2^ values from OptiQuant were assessed using GraphPad Prism 10 and Microsoft Excel 16. The statistical power was determined with unpaired two-tailed parametric Student’s *t*-test, and *p* values of <0.05 indicated statistical significance. Error bars signify mean ± standard deviation. The Shapiro–Wilk test confirmed normality of distribution among all groups. Pearson’s correlation and linear regression were used for the parametric comparison between groups for age and [^125^I]α-Bgtx binding in DLU/mm^2^ for both PD and CN.

## 3. Results

### 3.1. PD Cases

**Figure 3:**
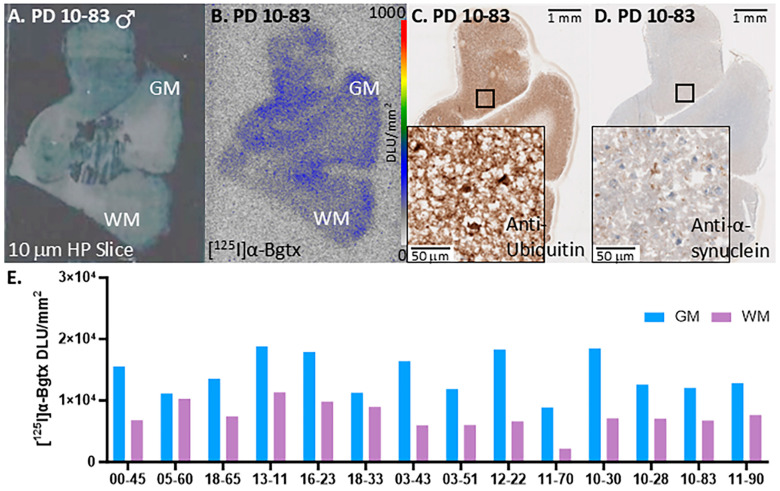
[^125^I]α-Bgtx binding to α7 nAChRs in representative male PD 10-83. (**A**). Postmortem human HP brain slices (10 μm) of male PD 10-83 mapping the distribution of GM and WM. (**B**). Autoradiographic image of PD 10-83 with [^125^I]α-Bgtx binding; autoradiography scale bar: 0–1000 DLU/mm^2^. (**C**). Anti-ubiquitin IHC image of PD 10-83 adjacent slice at 1 mm magnification; inset of IHC image at 50 mm magnification. (**D**). Anti-α-synuclein IHC image of PD 10-83 adjacent slice at 1 mm magnification; inset of 50 mm magnification. (**E**). [^125^I]α-Bgtx binding of all male PD in GM and WM.

All male PD cases were positively immunostained, visually confirming positivity for ubiquitin and α-synuclein as seen in male representative case PD 10-83 (Figure 3C,D). There was similar distribution of ubiquitin and α-synuclein among all cases as indicated by their IHC images. The ubiquitin and α-synuclein distribution were greater in GM than WM regions. The autoradiographic image of an adjacent slice shows binding of [^125^I]α-Bgtx to α7 nAChRs, which also was distributed within GM (Figure 3B). All cases had higher GM binding compared to the nonspecific binding in the WM. However, the ratios of GM/WM across all the subjects varied reflecting differences in the brain sections of each case.

**Figure 4:**
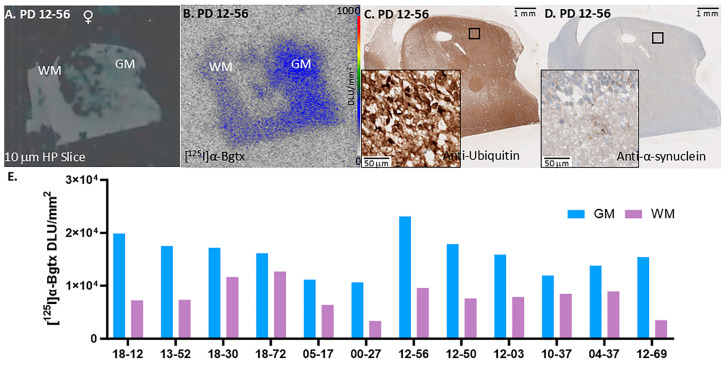
[^125^I]α-Bgtx binding to α7 nAChRs in representative female PD 12-56. (**A**). Postmortem human HP brain slices (10 μm) of female PD 12-56 mapping the distribution of GM and WM. (**B**). Autoradiographic image of PD 12-56 with [^125^I]α-Bgtx binding; autoradiography scale bar: 0–1000 DLU/mm^2^. (**C**). Anti-ubiquitin IHC image of PD 12-56 adjacent slice at 1 mm magnification; inset of IHC image at 50 mm magnification. (**D**). Anti-α-synuclein IHC image of PD 12-56 adjacent slice at 1 mm magnification; inset of 50 mm magnification. (**E**). [^125^I]α-Bgtx binding of all female PD in GM and WM.

All female PD cases were also confirmed to be positive for ubiquitin and α-synuclein in a similar distribution as female representative case PD 12-56 (Figure 4C,D). The autoradiograph of adjacent brain slices showed extensive [^125^I]α-Bgtx binding to α7 nAChRs in more GM regions than WM (Figure 4B,E). Similarly to the male cases, all female cases had higher GM binding compared to the nonspecific binding in the WM. The ratios of GM/WM across all the female subjects varied reflecting differences in the brain sections of each case.

### 3.2. CN Cases

**Figure 5:**
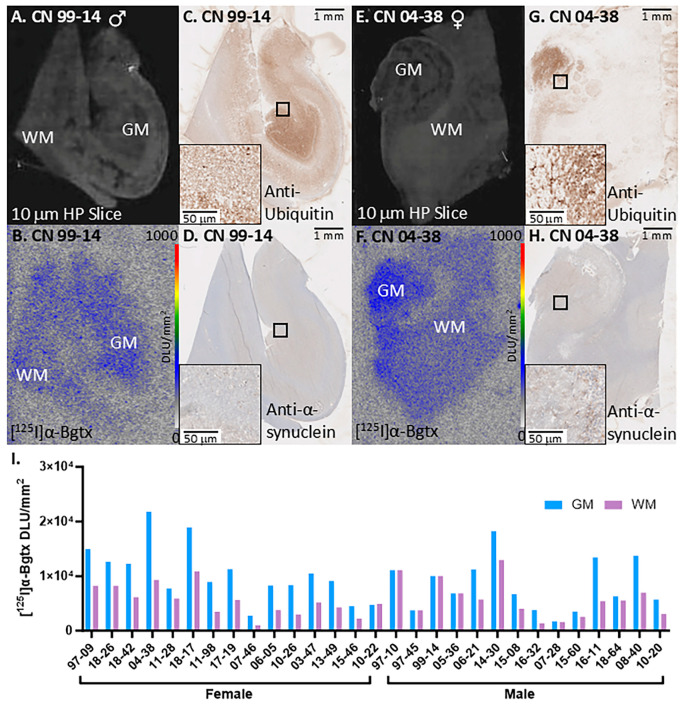
[^125^I]α-Bgtx binding to α7 nAChRs in representative CN 99-14 and 04-38. (**A**). Postmortem human HP brain slices (10 μm) of male CN 99-14 mapping the distribution of GM and WM. (**B**). Autoradiographic image of CN 99-14 with [^125^I]α-Bgtx binding; autoradiography scale bar: 0–1000 DLU/mm^2^. (**C**). Anti-ubiquitin IHC image of CN 99-14 adjacent slice at 1 mm magnification; inset of IHC image at 50 mm magnification. (**D**). Anti-α-synuclein IHC image of CN 99-14 adjacent slice at 1 mm magnification; inset of 50 mm magnification. (**E**). Postmortem human HP brain slices (10 μm) of male CN 04-38 mapping the distribution of GM and WM. (**F**). Autoradiographic image of CN 04-38 with [^125^I]α-Bgtx binding; autoradiography scale bar: 0–1000 DLU/mm^2^. (**G**). Anti-ubiquitin IHC image of CN 04-38 adjacent slice at 1 mm magnification; inset of IHC image at 50 mm magnification. (**H**). Anti-α-synuclein IHC image of CN 04-38 adjacent slice at 1 mm magnification; inset of 50 mm magnification. (**I**). [^125^I]α-Bgtx binding of all CN in GM and WM.

All CN cases were immunostained and visualized minimal ubiquitin and α-synuclein as seen in the representative cases CN 99-14 and 04-38 (Figure 5C,D,G,H). Autoradiographs of adjacent slices displayed [^125^I]α-Bgtx binding to α7 nAChRs (Figure 5B,F). Some cases shared similar [^125^I]α-Bgtx binding between GM and WM regions while others showed more within GM (Figure 5I). Similarly to the PD cases, there was a wide variation in the extent of [^125^I]α-Bgtx binding in the CN cases. This is likely due to the variation in the hippocampal sections across the CN cases.

### 3.3. Group Comparisons

**Figure 6:**
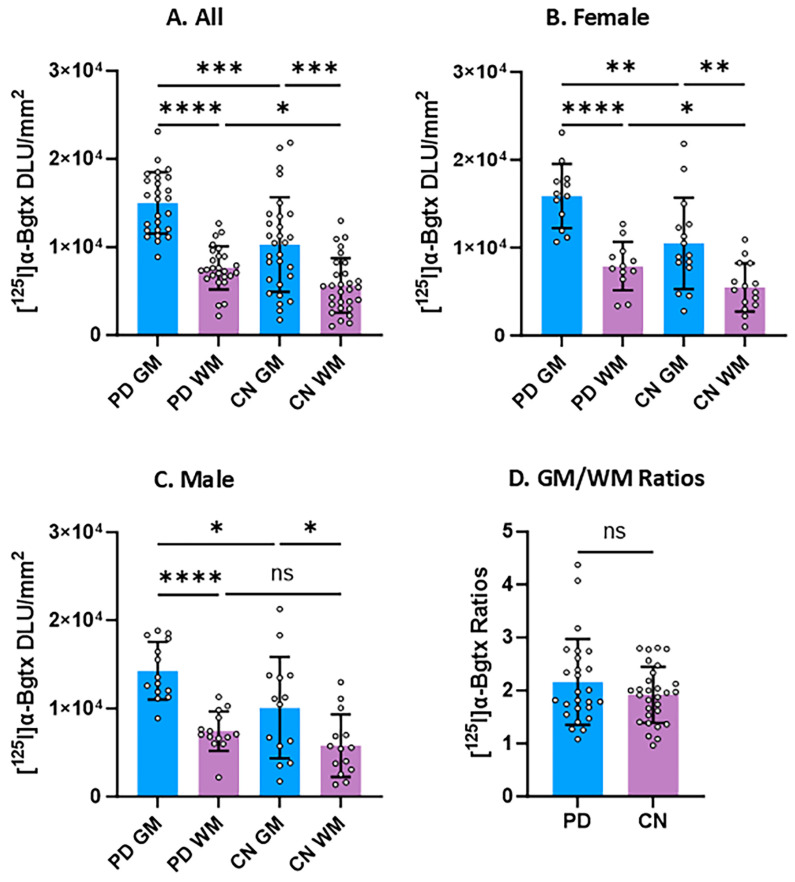
Comparisons between PD and CN in [^125^I]α-Bgtx binding. Unpaired two-tailed parametric *t*-tests determined statistical significance between each parameter (* *p* < 0.05, ** *p* < 0.01, *** < 0.001, **** < 0.0001, ns = not significant). (**A**). [^125^I]α-Bgtx binding in GM and WM of all male and female subjects. (**B**). GM and WM [^125^I]α-Bgtx binding in all female subjects. (**C**). GM and WM [^125^I]α-Bgtx binding in all male subjects. (**D**). [^125^I]α-Bgtx GM/WM ratios of PD and CN.

With regard to [^125^I]α-Bgtx binding in GM and WM, several comparisons were performed between PD and CN cases. Differences between PD cases and CN cases were highly significant. The greatest significance was in the GM regions, while even the WM was found to be different. There was an increase of 32% in [^125^I]α-Bgtx binding in all PD GM compared to all CN GM. Female PD GM showed a 34% increase compared to female CN GM (Figure 6B). Among male cases, PD GM was significantly greater than male CN GM by 29% (Figure 6C). Figure 6D shows the [^125^I]α-Bgtx GM/WM ratios with a difference of 11% between PD and CN.

**Figure 7:**
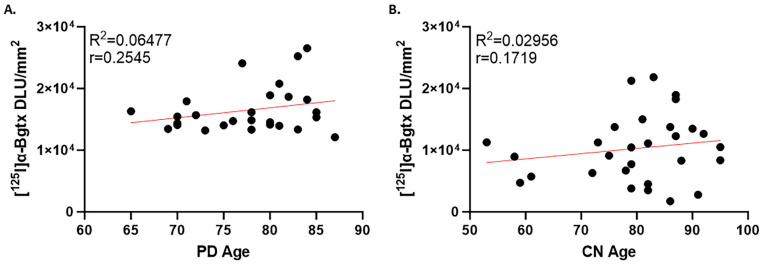
Comparisons between PD and CN in [^125^I]α-Bgtx binding. (**A**). [^125^I]α-Bgtx binding in GM correlated to age in PD cases (Pearson’s r = 0.2545). (**B**). [^125^I]α-Bgtx binding in GM correlated to age in CN cases (Pearson’s r = 0.1719).

To observe any potential connections between α7 nAChRs and age, [^125^I]α-Bgtx binding in the GM was correlated with the age for each case in PD (Figure 7A) and CN (Figure 7B). PD cases in this study spanned over three decades while CN spanned over five decades. Although both groups exhibited poor Pearson’s correlations, there was an upward trend with aging suggesting no loss of α7 nAChRs.

**Figure 8:**
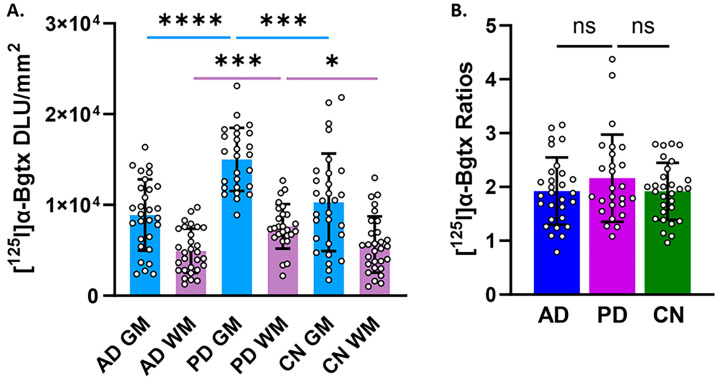
Comparisons between AD, PD, and CN in [^125^I]α-Bgtx binding. Unpaired two-tailed parametric *t*-tests determined statistical significance between each parameter (* *p* < 0.05, *** < 0.001, **** < 0.0001, ns = not significant). (**A**). [^125^I]α-Bgtx binding in GM and WM in AD, PD, and CN cases. (**B**). [^125^I]α-Bgtx GM/WM ratios.

We previously reported the findings on [^125^I]α-Bgtx binding in AD hippocampus postmortem brain slices [9]. It is of interest to compare the distribution of α7 nAChRs in different neurodegenerative diseases such as AD and PD within the same brain region. Figure 8A shows that PD GM is significantly greater than AD GM by 41%. The GM/WM ratios of PD is greater than AD by 11% but this difference is not statistically significant (Figure 8B). Spillover effects from the GM to WM cannot be ruled out and other image analysis issues may be causing the lower GM/WM ratio in PD. Thus, a ratio analysis in these small brain sections due to the high variability in the binding to WM as seen in Figure 3, Figure 4 and Figure 5 may lead to underestimation of the differences between AD and PD.

## 4. Discussion

Various signaling pathways throughout the brain such as the hippocampus include α7 nAChRs, playing crucial roles in cholinergic neurotransmission. However, these pathways can be compromised in neurodegeneration such as in PD. As a standard radioligand for α7 nAChRs, [^125^I]α-Bgtx was used to assess how α7 nAChR expression is affected in PD. The results in this study suggest that [^125^I]α-Bgtx is selective to α7 nAChRs in GM regions of postmortem hippocampus brain slices of PD. The distribution of [^125^I]α-Bgtx binding to α7 nAChRs generally aligned with ubiquitin and α-synuclein distribution in IHC images. Significant differences were observed between PD and CN in [^125^I]α-Bgtx binding and visual presence of ubiquitin and α-synuclein. Age correlation plots with [^125^I]α-Bgtx binding in PD and CN showed a weak positive association. The amount of [^125^I]α-Bgtx binding to PD cases significantly differed from the AD cases that were previously reported [9].

Interestingly, our results show an increase in α7 nAChR expression among PD cases compared to CN cases. An [^18^F]ASEM study observed an early increase in expression first occurring in the striatum of a 6-hydroxydopamine-lesioned rats simulating PD [28]. A potential explanation for this observed increase can be attributed to nAChR-mediated protection against neurotoxicity induced by 6-hydroxydopamine [39]. In monkeys treated with MPTP, there was also an increase in α7 nAChR expression [29]. This may be a compensatory mechanism that also involves protection against cell damage and apoptosis induced by α-synuclein, promoting the clearance of α-synuclein [40]. Earlier stages of PD may attempt to maintain cell integrity with compensatory mechanisms that increase α7 nAChR expression before becoming overwhelmed and eventually decreasing with motor and cognitive function. An initial increase and then decrease in α7 nAChR expression may align with studies that observe only a depletion or no significant differences [41,42,43]. The expression level may differ depending on the severity of PD and the region observed. However, these results agree upon the potential of α7 nAChRs as neuroprotective therapeutic targets in PD.

The brains of CN individuals may have Lewy bodies due to Lewy body disease but also because of normal aging, both of which are affected by greater vulnerability of mitochondrial dysfunction in the hippocampus, substantia nigra, and amygdala [44]. In this study, there was minimal presence of ubiquitin and α-synuclein in the IHC images, so it is unlikely that they substantially affected α7 nAChR expression. When comparing CN GM and WM to PD, standard deviation was greater and therefore had more variety in [^125^I]α-Bgtx binding. The same CN cases were previously compared to AD cases which had similar variety in [^125^I]α-Bgtx binding but had no significant differences from CN [9]. Both neurodegenerative diseases impact the same receptors but the specific mechanisms involving these receptors may differ. Activation of nAChRs induces neuroprotection against glutamate toxicity in AD and against α-synuclein toxicity and dopaminergic neuron loss in PD [39,45]. Neuroprotective pathways include nAChRs mediating microglial Aβ clearance, which is therapeutically relevant to AD, while the PI3K/AKT and JAK2/STAT3 pathways prevent apoptosis and neuroinflammation, primarily important in PD [39]. Differences in α7 nAChR expression in separate neurodegenerative diseases such as AD and PD can indicate roles that are unique to individual conditions.

Studies using [^125^I]α-Bgtx binding measure changes in the levels of α7 nAChR expression. They do not, however, measure the functional state of α7 nAChR. It is plausible that PD and AD pathologies may affect the ion channel without affecting protein expression. For example, abnormalities in the functional state of the N-methyl-D-aspartate (NMDA) ion channel in AD have been found, without necessarily affecting its expression levels [46]. This overactive NMDA ion-channel in AD has therefore been a target for therapeutic drugs such as memantine [46]. Other G-protein coupled receptors such as serotonin 5HT1A and dopamine D2 receptor have shown a small increase or no change in the anterior cingulate of PD subjects [47]. Although the compromised integrity of the pathways involving such receptors is known to contribute to neuropathological features of PD, measurements of expression and function may misalign. The downstream effects of the alterations in receptor functions are measured using glucose metabolism studies by [^18^F] fluorodeoxyglucose (FDG)-PET in human PD [48,49,50,51,52]. These effects can be further specified when considering metabolic changes due to age and gender in PD. Although males are more commonly diagnosed with PD, diagnosis for females is more difficult to obtain due to a persistently younger metabolic brain age compared to chronological age during aging [53]. When adjusting for confounding factors influenced by age and gender on [^18^F]FDG-PET images, differential diagnosis of PD improved in specificity and sensitivity [53]. In this study, there were no obvious distinctions in age and gender differences in the context of [^125^I]α-Bgtx binding to α7 nAChRs but their functional state may indicate otherwise. Neurodegenerative diseases such as PD and AD can benefit from exploring how the impaired cholinergic system leads to neuropathological consequences. Other nAChR subtypes such as a4b2 can be investigated in tandem with common pathologies and potential differences in age and gender [33,54,55,56]. Specific patterns of cognitive and metabolic deficits in the PD brain can be identified and used for understanding PD.

Limitations of the study include adjacent brain slice variability during the assay conditions. Because hippocampal brain slices were used, the complete hippocampus-subiculum brain region may not have been adequately visualized or differentiated. Additionally, all PD cases had similar Lewy body stages so future studies will need to investigate potential differences in different stages and severity of PD. As a follow-up to our previous paper on [^125^I]α-Bgtx binding to hippocampal α7 nAChRs in AD, it was of interest in this study to observe if there were parallels to be made between the two neurodegenerative diseases [9]. Observations of [^125^I]α-Bgtx binding in PD alongside AD in this paper are preliminary and require supplementary experiments such as saturation binding curves to draw direct comparisons. Overall, these limitations did not hinder this study in validating [^125^I]α-Bgtx in selectively binding to α7 nAChRs in PD. In agreement with previous studies that accentuate the minimal off-target binding of [^125^I]α-Bgtx in several brain regions and neuron cultures, [^125^I]α-Bgtx is also selective to α7 nAChRs in postmortem human hippocampus [57,58]. Cognitive and motor function is substantially reliant on the activation of α7 nAChRs in neuroprotective pathways throughout the brain including the hippocampus. Expanding upon current understandings of mechanisms involving specific nAChR subtypes can be achieved with the development and implementation of radiotracers that may provide functional information.

## 5. Conclusions

Our results demonstrate that [^125^I]α-Bgtx is a viable radioligand for studies of human α7 nAChRs in PD hippocampus. Compared to CN cases, there was a small, but significant increase in PD. These findings seem consistent with previous studies of this receptor system in PD models. PET studies of α7 nAChR in PD subjects may be useful in further delineating the role of this important receptor system within multiple symptoms of PD. Alongside the α4β2 nAChRs, an understanding of the neuropathological consequences on the cholinergic system in the hippocampus is vital for PD and AD.

## Figures and Tables

**Table 1:** Patient samples and data *.

Cases, n	CERAD Pathology ^1^	Gender	Age Range, Mean ± SD	PMI, Hrs ^2^	Brain Region ^3^	Plaque Total	Tangle Total	LB ^4^	Braak Score
12	PD	Male	69–85 (76.8 ± 5.03)	1.83–4.37	HP	0–1.5	0–6.5	III–IV	II–IV
14	PD	Female	65–87 (78.1 ± 6.84)	2–5	HP	0–13	0.5–6.5	III–IV	I–IV
14	CN	Male	61–90 (79.3 ± 7.44)	2–5.42	HP	0–5.5	0–6	0	I–III
15	CN	Female	53–95 (80.3 ± 13.5)	2.07–4.83	HP	0–10	0.5–6.5	0–II	I–III

* Frozen brain samples were obtained from Banner Sun Health Institute, Sun City, Arizona described previously [37]. ^1^ CN = cognitively normal and may include mild cognitive impairment (MCI); PD = Parkinson’s disease; ^2^ PMI: postmortem interval in hours; ^3^ HP: hippocampus; ^4^ LB = Lewy bodies, quantified by Unified LB Stage I-IV [38]. Brain slices (10 μm thickness) were obtained from frozen tissue on a Leica 1850 cryotome cooled to −20 °C and collected on Fisher slides.

## Data Availability

The original contributions presented in this study are included in the article. Further inquiries can be directed to the corresponding author(s).
